# Genomic Analysis of the Appearance of Ovarian Mast Cells in Neonatal MRL/MpJ Mice

**DOI:** 10.1371/journal.pone.0100617

**Published:** 2014-06-23

**Authors:** Teppei Nakamura, Yuko Sakata, Saori Otsuka-Kanazawa, Osamu Ichii, Masataka Chihara, Ken-ichi Nagasaki, Yuka Namiki, Yasuhiro Kon

**Affiliations:** 1 Laboratory of Anatomy, Department of Biomedical Sciences, Graduate School of Veterinary Medicine, Hokkaido University, Sapporo, Hokkaido, Japan; 2 Section of Biological Safety Research, Chitose Laboratory, Japan Food Research Laboratories, Chitose, Hokkaido, Japan; 3 Office for Faculty Development and Teaching Enriched Veterinary Medicine, Graduate School of Veterinary Medicine, Hokkaido University, Sapporo, Hokkaido, Japan; John Hopkins University School of Medicine, United States of America

## Abstract

In MRL/MpJ mice, ovarian mast cells (OMCs) are more abundant than in other mouse strains, and tend to distribute beneath the ovarian surface epithelium at birth. This study investigated the factors regulating the appearance of neonatal OMCs in progeny of the cross between MRL/MpJ and C57BL/6N strains. F1 neonates had less than half the number of OMCs than MRL/MpJ. Interestingly, MRLB6F1 had more neonatal OMCs than B6MRLF1, although they were distributed over comparable areas. Furthermore, in MRL/MpJ fetuses for which parturition was delayed until embryonic day 21.5, the number of OMCs was significantly higher than in age-matched controls at postnatal day 2. These results suggest that the number of OMCs was influenced by the environmental factors during pregnancy. Quantitative trait locus analysis using N2 backcross progeny revealed two significant loci on chromosome 8: *D8Mit343*–*D8Mit312* for the number of OMCs and *D8Mit86*–*D8Mit89* for their distribution, designated as *mast cell in the ovary of MRL/MpJ 1* (*mcom1*) and *mcom2*, respectively. Among MC migration-associated genes, ovarian expression of chemokine (C-C motif) ligand 17 at *mcom1* locus was significantly higher in MRL/MpJ than in C57BL/6N, and positively correlated with the expression of OMC marker genes. These results indicate that the appearance of neonatal OMCs in MRL/MpJ is controlled by environmental factors and filial genetic factors, and that the abundance and distribution of OMCs are regulated by independent filial genetic elements.

## Introduction

Mast cells (MCs) reside in most tissues and act as sentinel cells in both innate and adaptive immunity [1]. In addition to immunological processes such as allergy and autoimmunity, MCs also contribute to the pathogenesis of cancer, obesity, and diabetes [2]–[4]. The number of MCs in reproductive organs varies over the estrous cycle, suggesting an important role in reproductive function under the control of steroid hormones [5], [6]. However, MCs are present even in the neonatal mouse ovary [7]. In ICR mice, MCs in the ovarian hilus, mesovarium, and ovarian bursa are most abundant at postnatal day 0 (P0), with numbers decreasing during the first postnatal week.

MRL/MpJ mice originate from C57BL/6J (0.3%), C3H/HeDi (12.1%), AKR/J (12.6%), and LG/J (75.0%) strains. MRL/MpJ mice and their mutant strain, MRL/MpJ-*lpr/lpr* mice, are models for autoimmune diseases that resemble human systemic lupus erythematosus and rheumatoid arthritis [8], [9]. In addition to autoimmune diseases, MRL/MpJ mice show some unique phenotypes related to wound healing, such as accelerated ear punch closure and cardiomyocyte regeneration [10], [11]. We previously reported other unique characteristics in the reproductive organs of MRL/MpJ mice; *i.e.*, metaphase-specific apoptosis of meiotic spermatocytes, heat shock resistance of spermatocytes found in experimental cryptorchidism, existence of testicular oocytes in newborn males, and development of ovarian cysts originating from the rete ovarii [12]–[15]. These phenotypes were closely associated with the genetic background of MRL/MpJ mice, and several susceptibility loci were identified by quantitative trait locus (QTL) analysis [16]–[20].

We have observed that neonatal MRL/MpJ mice possess numerous MCs in the ovary compared to other inbred mouse strains including C57BL/6N [21]. In addition, there is a population of ovarian MCs (OMCs) localized beneath the ovarian surface epithelium (SE) in neonatal MRL/MpJ mice, which has a possible association with early follicular development. In the present study, the factors affecting the appearance of OMCs in neonatal MRL/MpJ mice were investigated.

## Materials and Methods

### Ethical statement

This study was approved by the Institutional Animal Care and Use Committee convened at the Graduate School of Veterinary Medicine, Hokkaido University (approval number: 13-0086). The investigators adhered to the Guide for the Care and Use of Laboratory Animals of Hokkaido University, Graduate School of Veterinary Medicine (approved by the Association for the Assessment and Accreditation of Laboratory Animal Care International).

### Animals

MRL/MpJ and C57BL/6N mice purchased from Japan SLC (Hamamatsu, Shizuoka, Japan) were used in this study. Timed mating was established by housing females with males overnight. At noon of the following day, females were checked for the presence of a vaginal plug, and the embryos were recorded as embryonic day 0.5 (E0.5). C57BL/6N mice were mated with MRL/MpJ mice to produce F1 and F2 progeny. In addition, 200 female N2 backcross progeny (BMM) were generated for QTL analysis by mating female B6MRLF1 (a cross between female C57BL/6N and male MRL/MpJ mice) with male MRL/MpJ mice.

### Histological analysis and phenotyping

Collected ovaries were fixed in 4% paraformaldehyde overnight, embedded in paraffin, and cut into 3-µm sections. Deparaffinized sections were stained with 1% toluidine blue in 70% ethanol for 30 min. The number of metachromatic MCs per total ovarian area was measured as the OMC density (cells/mm^2^) as previously described [21]. The number of MCs facing the ovarian SE per total ovarian area was measured as the SEMC density (cells/mm^2^) [21]. The SEMC ratio was calculated as the ratio of SEMC to OMC densities.

### Artificially delayed parturition model

To delay parturition, pregnant MRL/MpJ and C57BL/6N mice were subcutaneously injected with progesterone (2 mg/body; Sigma-Aldrich, St. Louis, USA) suspended in sesame oil (20 mg/ml) every 24 h from E17.5 to E20.5 [22]. At E21.5 when the parturition was delayed for 2 days, ovaries of fetuses were collected, with those of age-matched P2 mice serving as controls. P0 and P4 ovaries were also examined.

### Measurement of plasma sex hormone levels

Heparinized plasma was collected from female fetuses of MRL/MpJ and C57BL/6N mice at E18.5 of normal pregnancy. Plasma estradiol and progesterone were measured by enzyme-linked immunosorbent assay (Cayman Chemical Company, Ann Arbor, USA).

### Genotyping

Genomic DNA was prepared from the tail of each BMM mouse using a standard protocol. In total, 94 microsatellite markers identified in the Mouse Genome Informatics database of the Jackson Laboratory (www.informatics.jax.org) were used for a genome-wide scan, with a mean intermarker distance of 10–20 cM, to genotype the C57BL/6N or MRL/MpJ allele (Table S1). PCR was performed with GoTaq (Promega, Madison, USA) under the following PCR conditions: 2 min at 94°C; 40 cycles of 40 s at 94°C, 30 s at 58°C, and 20 s at 72°C; and 5 min at 72°C. To compare sizes of PCR products, amplified samples were resolved by electrophoresis using 2–4% agarose 3∶1 (AMRESCO, Inc., Solon, USA) which was stained with ethidium bromide and imaged under an ultraviolet lamp.

### QTL analysis

To examine susceptibility loci associated with the appearance of OMCs in neonatal MRL/MpJ mice, QTL analysis was performed using 200 BMM mice at P0. For the appearance of MCs in neonatal ovary, OMC density, SEMC density, and SEMC ratio were applied as quantitative traits (QT). Linkage analyses were performed using the Map Manager QTX20b program [23]. Thresholds for likelihood ratio statistics (LRS) were determined by genome-wide 1000-permutation test to provide the suggestive, significant, and highly significant levels. In addition, epistatic interactions between pairs of marker loci were also searched using the Map Manager software. The threshold for significant LRS was estimated by a 1000-permutation test.

### In silico analysis of single nucleotide polymorphisms

For several genes coded as highly significant QTL, single nucleotide polymorphisms (SNPs) specifically associated with non-synonymous coding polymorphisms were determined in MRL/MpJ- and C57BL/6N-type genomes using the Mouse Phenome Database (MPD) from Jackson Laboratory (http://phenome.jax.org).

### Reverse transcription and quantitative real-time PCR

Total RNA from ovaries of P0 MRL/MpJ and C57BL/6N mice was purified using TRIzol reagent (Life Technologies, Carlsbad, USA) and treated with DNase (Nippon Gene, Tokyo, Japan). Complementary DNA (cDNA) was synthesized from the RNA using ReverTra Ace (Toyobo, Osaka, Japan) and random primers (Promega). Each cDNA, adjusted to 1.0 µg/µL, was used for quantitative real-time PCR (qPCR) with Brilliant III SYBR Green QPCR Master Mix (Agilent, Santa Clara, USA). Gene-specific primer pairs are listed in Table S2.

### Statistical analysis

Results are expressed as mean ± SEM and were analysed using nonparametric methods. The Mann-Whitney *U* test was used to compare two groups. The Kruskal-Wallis test was used for comparisons of more than two groups, and multiple comparisons were performed using Scheffé’s method. Correlations were evaluated using Pearson’s correlation test.

## Results

### Appearance of OMCs in MRL/MpJ and C57BL/6N mice, and their progeny

**Figure 1:**
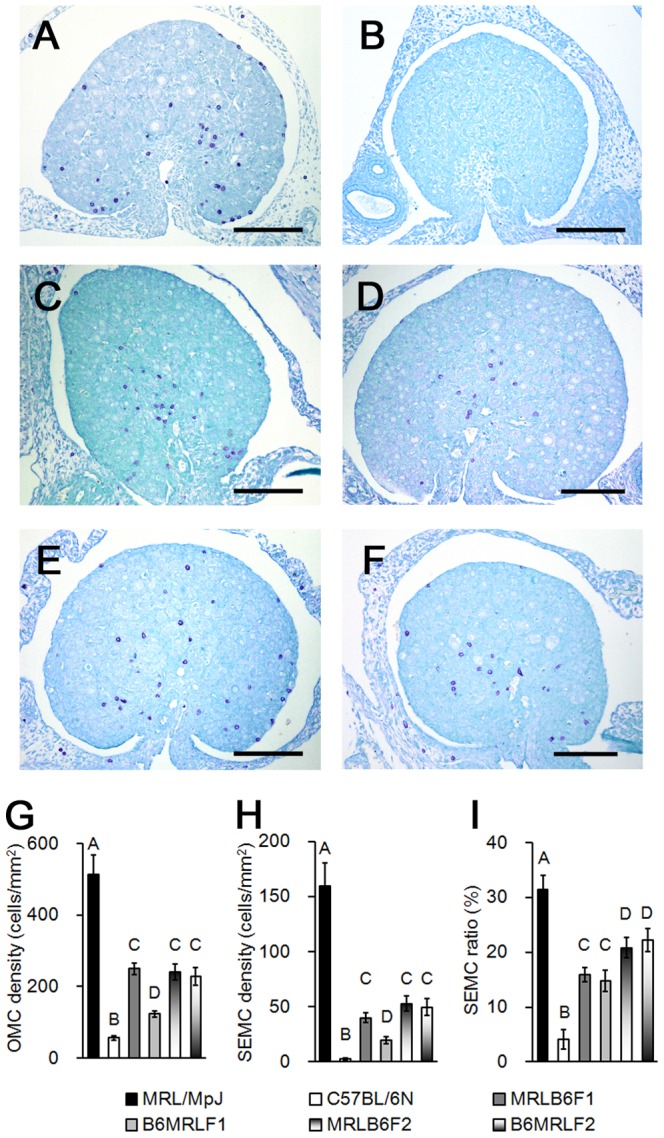
Appearance of ovarian mast cells in MRL/MpJ and C57BL/6N mice, and F1 and F2 intercross mice at postnatal day 0. (A–F) Toluidine blue-stained sections of ovaries in (A) MRL/MpJ, (B) C57BL/6N, (C) MRLB6F1, (D) reciprocal F1 (B6MRLF1), (E) MRLB6F2, and (F) reciprocal F2 (B6MRLF2). Bars: 100 µm. (G) Number of mast cells per total ovarian area (OMC density). (H) Number of mast cells facing ovarian surface epithelium per total ovarian area (SEMC density). (I) Ratio of SEMC density to OMC density (SEMC ratio). Data represent mean ± SEM (n = 10–20 per group). Significant differences were analysed using Scheffé’s method following the Kruskal-Wallis test. Statistical significance is indicated by different letters (*P* < 0.05).

To determine the pattern of inheritance, the appearance of OMCs was compared between MRL/MpJ and C57BL/6N mice and their F1 and F2 progeny at P0. Metachromatic MCs were abundant in ovaries of MRL/MpJ mice (Fig. 1A), but rarely seen in C57BL/6N ovaries (Fig. 1B). OMCs were observed in both direct F1 (MRLB6F1, a cross between female MRL/MpJ and male C57BL/6N mice) and reciprocal F1 (B6MRLF1) at P0 (Fig. 1C, D). The F2 progeny (MRLB6F2 and B6MRLF2) also had OMCs at P0 (Fig. 1E, F). Although MCs were distributed in the deep layer of the ovarian cortex in MRL/MpJ, F1, and F2 mice, they were more frequently observed beneath the ovarian SE in MRL/MpJ mice than in their F1 and F2 progeny (Fig. 1A, C–F).

OMC density was 50-fold higher in MRL/MpJ than in C57BL/6N mice (Fig. 1G), while values in F1 and F2 were less than half of that observed in the MRL/MpJ parental strain. These results suggested that the appearance of OMCs was controlled by at least recessive factor. Notably, MRLB6F1 mice had a significantly higher number of OMCs than B6MRLF1 (Fig. 1G). In contrast, no differences were observed in OMC density between MRLB6F2 and B6MRLF2. Similar trends were observed for SEMC density (Fig. 1H). The SEMC ratio, an index of OMC localization beneath the ovarian SE, was also higher in MRL/MpJ than in C57BL/6N, F1, and F2; however, the ratio was similar between direct and reciprocal F1 progeny (Fig. 1I). These results suggest that both parental strains and filial genetic factors affect the abundance of OMCs, but distribution of MCs beneath the ovarian SE is mainly regulated by filial genetic factors.

### Effects of maternal factors on the appearance of OMC

**Figure 2:**
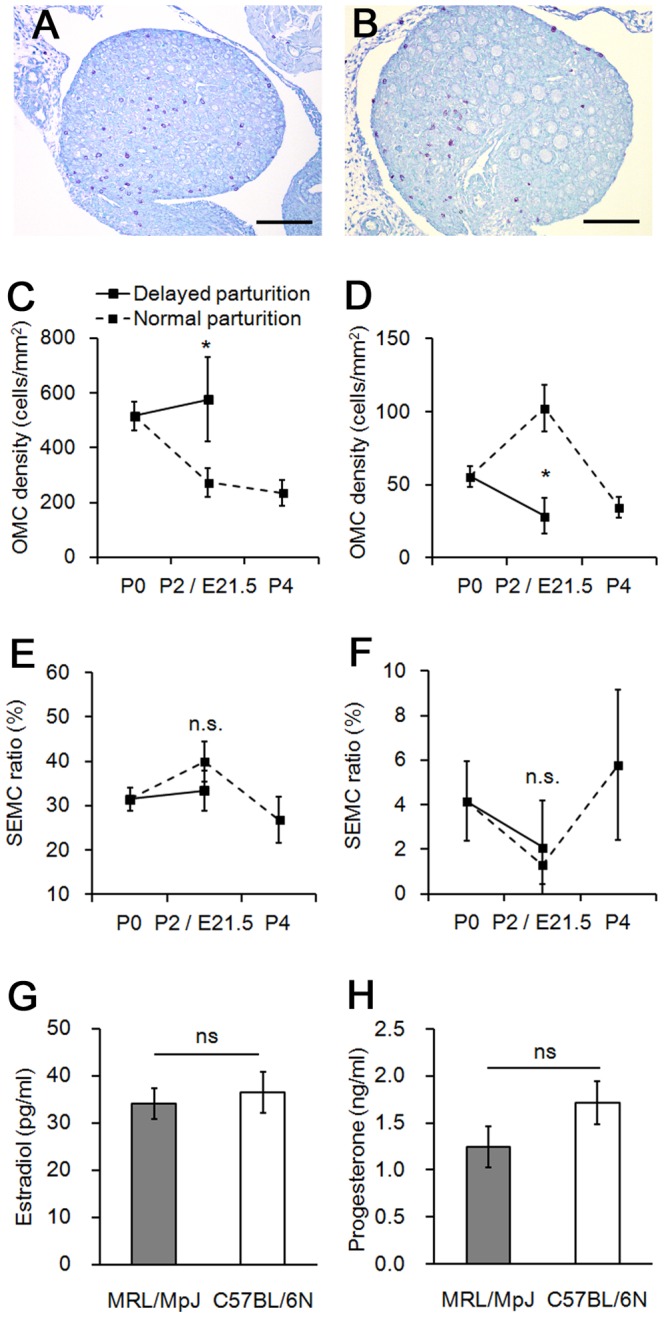
Effect of delayed parturition on appearance of ovarian mast cells in MRL/MpJ and C57BL/6N mice. (A and B) Toluidine blue-stained sections of MRL/MpJ mice ovaries at (A) embryonic day 21.5 with artificially delayed parturition, and (B) postnatal day 2. Bars: 100 µm. (C) Number of mast cells per total ovarian area (OMC density) in MRL/MpJ mice. (D) OMC density in C57BL/6N mice. (E) Ratio of number of mast cells facing ovarian surface epithelium per total ovarian area to OMC density (SEMC ratio) in MRL/MpJ mice. (F) SEMC ratio in C57BL/6N mice. (G) Plasma estradiol concentration at embryonic day 18.5. (H) Plasma progesterone concentration at embryonic day 18.5. Data represent mean ± SEM (n = 3–13 per group). Significant differences were analysed using the Mann-Whitney *U* test. *P < 0.05; ns, not significant.

Based on the results of histoplanimetric analyses of F1 progeny, it was supposed that environmental factors during pregnancy affect the abundance of OMCs in perinatal mice. To test this hypothesis, parturition was delayed by injecting pregnant MRL/MpJ and C57BL/6N mice with progesterone, and OMC density was compared between E21.5 fetuses and age-matched P2 pups. In MRL/MpJ mice, MCs were abundant in the ovary at E21.5 (Fig. 2A), and these numbers tended to be higher than in P2 ovary of normal parturition (Fig. 2B). OMC density in normal parturition mice was highest at P0, and gradually decreased thereafter, but in E21.5 fetuses, the value was higher than in age-matched P2 mice, and comparable to P0 mice of normal parturition (Fig. 2C). In contrast, in C57BL/6N mice, OMC density at E21.5 was lower than in P2 controls (Fig. 2D). Consistent with the F1 analysis, SEMC ratio did not differ between E21.5 fetuses and age-matched P2 mice in both strains (Fig. 2E, F). In mammals, fetuses are exposed to high doses of steroid hormones during late pregnancy, and their hormones affect MC properties in the adult ovary [24]. However, neither plasma estradiol nor progesterone levels differed between MRL/MpJ and C57BL/6N mice at E18.5 of normal pregnancy (Fig. 2G, H).

### QTL analysis of OMC appearance in neonatal BMM mice

**Figure 3:**
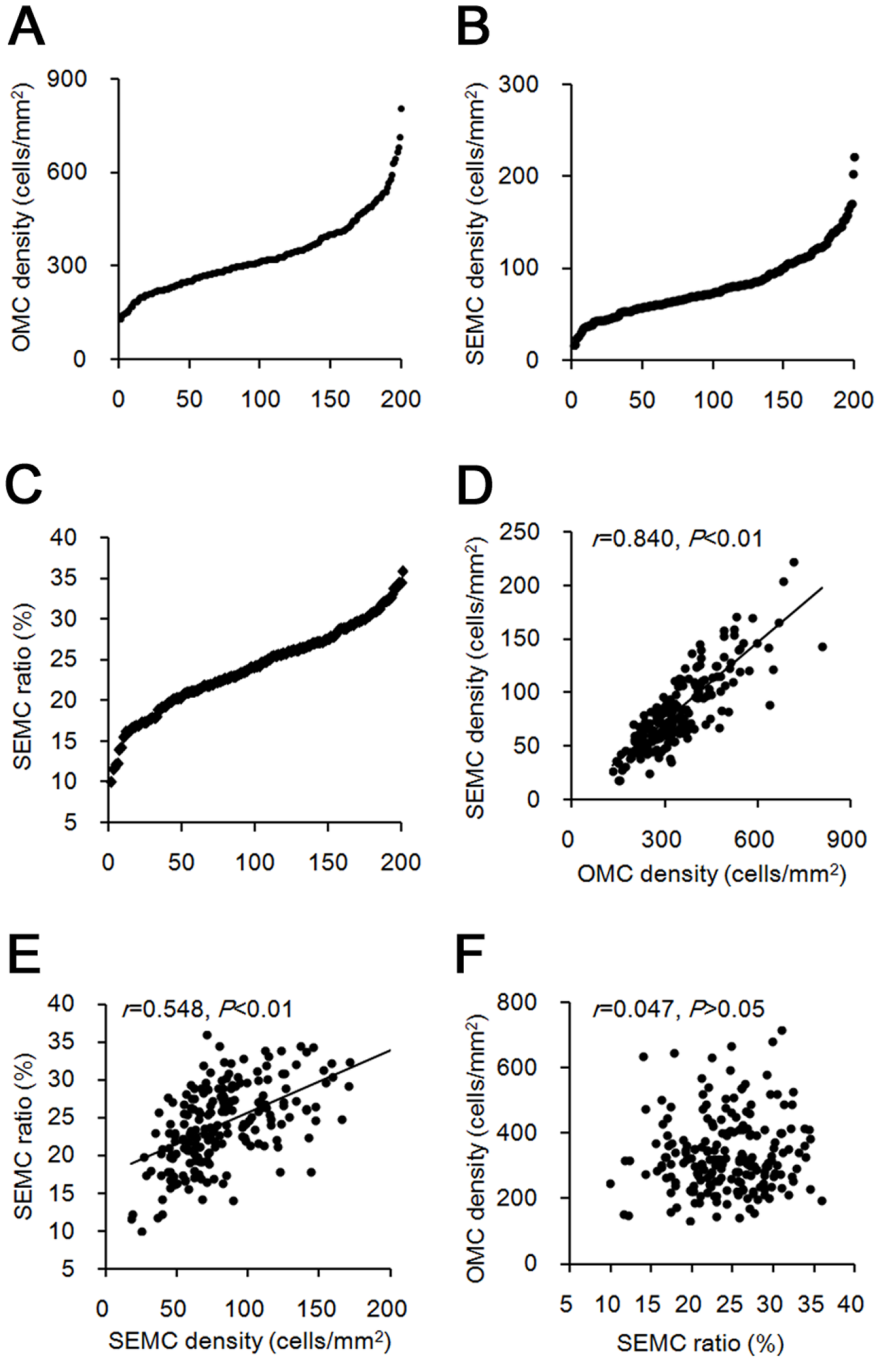
Appearance of ovarian mast cells in BMM progeny at postnatal day 0. (A) Distribution of number of mast cells per total ovarian area (OMC density). (B) Distribution of number of mast cells facing ovarian surface epithelium per total ovarian area (SEMC density). (C) Distribution of ratio of SEMC to OMC densities (SEMC ratio). (D) Correlation between OMC and SEMC densities (Pearson’s correlation test, n = 200). (E) Correlation between SEMC density and SEMC ratio (Pearson’s correlation test, n = 200). (F) Correlation between SEMC ratio and OMC density (Pearson’s correlation test, n = 200).

To identify the genetic loci controlling the appearance of OMCs in neonatal MRL/MpJ mice, a genome-wide QTL analysis was performed using 200 BMM mice at P0. Since both OMC abundance and their distribution beneath the ovarian SE were unique phenotypes of MRL/MpJ mice, OMC density, SEMC density, and SEMC ratio were measured as QTs. All QTs had gradient distributions, suggesting a polygenic pattern of inheritance (Fig. 3A–C). SEMC density was positively correlated with both OMC density and SEMC ratio (Fig. 3D, E), but there was no correlation between these latter traits (Fig. 3F), suggesting that OMC density and SEMC ratio were independent QTs corresponding to the number and distribution of OMCs, respectively, while SEMC density arose from the combination of these two traits.

**Figure 4:**
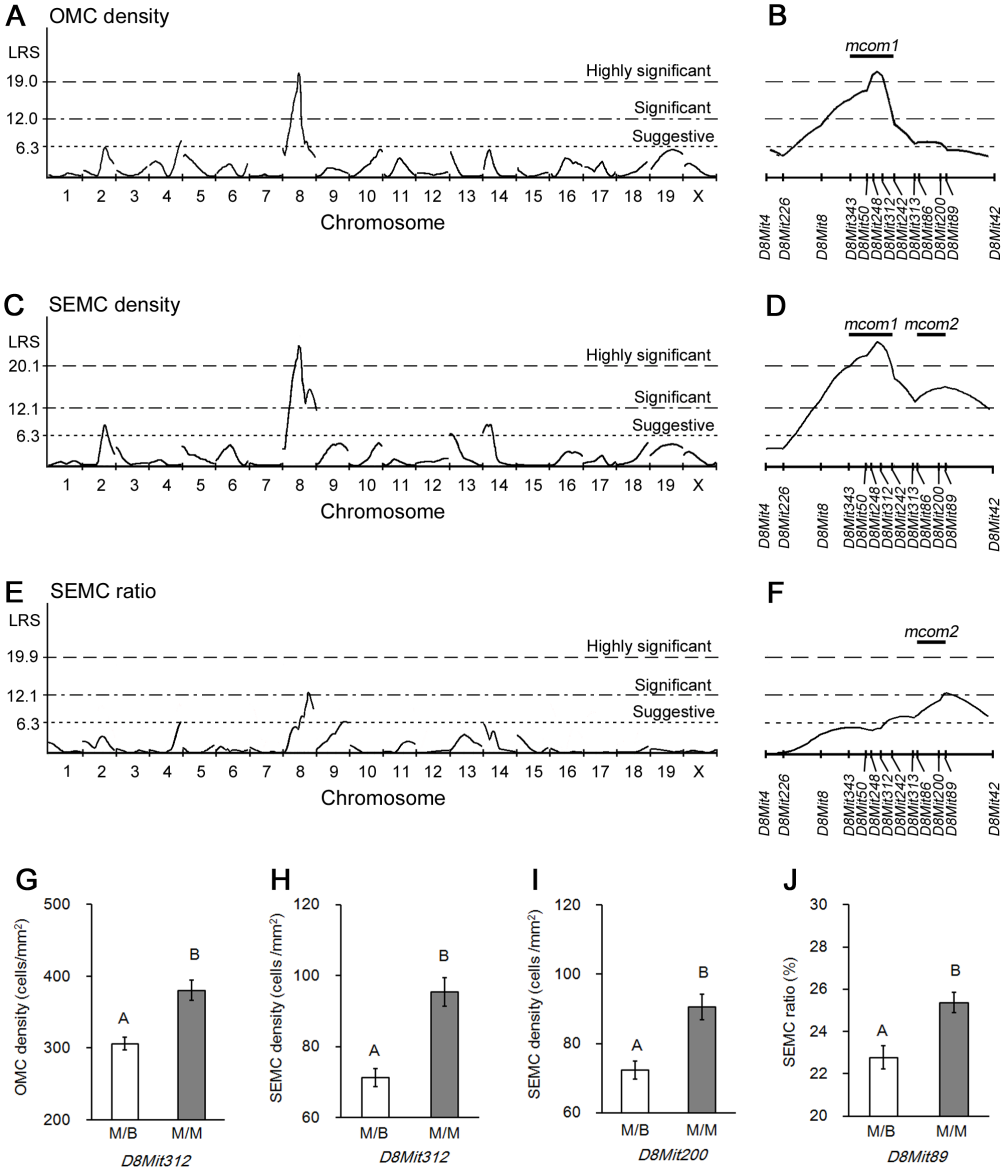
Quantitative trait loci linkage analysis for the appearance of ovarian mast cells. (A) Interval mapping for number of mast cells per total ovarian area (OMC density). (B) Interval mapping on Chr 8 for OMC density. (C) Interval mapping for number of mast cells facing ovarian surface epithelium per total ovarian area (SEMC density). (D) Interval mapping on Chr 8 for SEMC density. (E) Interval mapping for ratio of OMC to SEMC densities (SEMC ratio). (F) Interval mapping on Chr 8 for SEMC ratio. (G) Allele effect of *D8Mit312* on OMC density. (H) Allele effect of *D8Mit312* on SEMC density. (I) Allele effect of *D8Mit200* on SEMC density. (J) Allele effect of *D8Mit89* on SEMC ratio. M/B indicates mice heterozygous with both a MRL/MpJ and C57BL/6N allele. M/M indicates mice homozygous for the MRL/MpJ allele. Data represent mean ± SEM. Significant differences were analysed using the Mann-Whitney *U* test. Statistical significance is indicated by different letters (*P* < 0.01).

Using OMC density as a trait, suggestive QTL were mapped to C57BL/6N-type *D4Mit42* (LRS  =  7.0, 82.64 cM) (Fig. 4A, Table 1). In addition, a significant locus was mapped to MRL/MpJ-type *D8Mit343*–*D8Mit312* (LRS > 12.0, 39.33–47.12 cM), with one highly significant peak on *D8Mit248*–*D8Mit312* (LRS > 19.0, 44.99–47.12 cM) (Fig. 4B, G, Table 1).

**Table 1:** Location of QTL for ovarian mast cells in BMM backcross progeny.

			OMC density			SEMC density			SEMC ratio		
Chr.	Peak cM	Microsatellite Marker	LRS	% variance	*P* value	LRS	% variance	*P* value	LRS	% variance	*P* value
2	73.59	*D2Mit340*				8.4	4	0.00333			
4	82.64	*D4Mit42*	7.0	4	0.00754				6.6	3	0.00958
8	44.99	*D8Mit248*	20.4**	10	0.00001						
	47.12	*D8Mit312*	20.4**	10	0.00001	24.5**	12	<0.00001			
	62.93	*D8Mit89*				15.9*	8	0.00007	12.6*	6	0.00039
9	71.49	*D9Mit18*							6.4	3	0.01238
13	7.73	*D13Mit17*				6.5	3	0.01138			
14	16.80	*D14Mit133*				8.1	4	0.00509			

LRS scores above the suggestive level are shown. *significant; **highly significant. OMC density: number of mast cells per total ovarian area. SEMC density: number of mast cells facing ovarian surface epithelium per total ovarian area. SEMC ratio: ratio of SEMC to OMC densities.

For SEMC density, suggestive loci were mapped to MRL/MpJ-type *D2Mit340* (LRS  =  8.4, 73.59 cM), MRL/MpJ-type *D13Mit17* (LRS  =  6.5, 7.73 cM), and C57BL/6N-type *D14Mit11-D14Mit141* (LRS > 6.3, 6.33-24.28 cM) (Fig. 4C, Table 1). Bimodal, significant peaks were detected on Chr 8 (Fig. 4D). Significant QTL were mapped to MRL/MpJ-type *D8Mit8*–*D8Mit89* (LRS > 12.1, 32.30–62.93 cM), with one highly significant peak on *D8Mit343*–*D8Mit312* (LRS > 20.1, 39.33–47.12 cM) overlapping the highly significant locus for OMC density, and the other peak on *D8Mit86*–*D8Mit89* (LRS > 12.1, 56.18–62.93 cM) (Fig. 4D, H, I, Table 1). These two significant QTL were designated as *mast cell in the ovary of MRL/MpJ 1* (*mcom1*) and *mcom2*, respectively.

Suggestive QTL for SEMC ratio were mapped to C57BL/6N-type *D4Mit42* (LRS  =  6.6, 82.64 cM) and MRL/MpJ-type *D9Mit18* (LRS  =  6.4, 71.49 cM) (Fig. 4E, Table 1), while a significant QTL was mapped to MRL/MpJ-type *D8Mit89* (LRS  =  12.6, 62.93 cM), which overlapped with *mcom2* (Fig 4F, J, Table 1).

### Analysis of epistatic interactions involved in OMC appearance

Epistatic interactions were examined to determine QTL responsible for the appearance of OMCs. QTL interaction was identified between *D8Mit312* (47.12 cM) and *D6Mit74* (23.70 cM) for both OMC and SEMC densities. Additional QTL interactions were identified between *D8Mit248* (44.99 cM) and *D19Mit33* (51.76 cM) for SEMC density, and between *D8Mit89* (62.93 cM) and *D19Mit91* (40.53 cM) for SEMC ratio.

**Figure 5:**
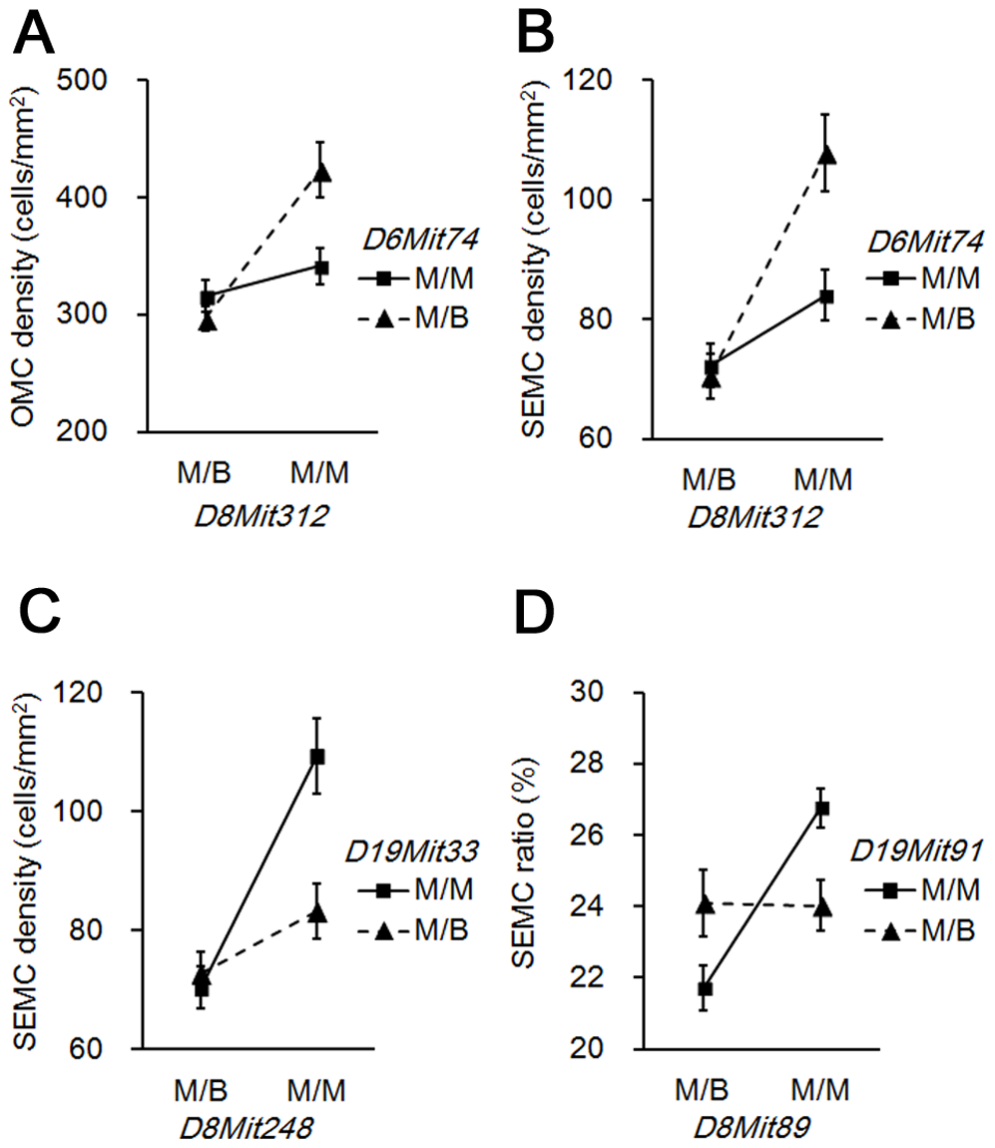
Epistatic interactions regulating the appearance of ovarian mast cells. (A) Genotypic interactions between *D8Mit312* and *D6Mit74* for the number of mast cells per total ovarian area (OMC density). (B) Genotypic interactions between *D8Mit312* and *D6Mit74* for the number of mast cells facing ovarian surface epithelium per total ovarian area (SEMC density) (C) Genotypic interactions between *D8Mit248* and *D19Mit33* for SEMC density. (D) Genotypic interactions between *D8Mit89* and *D19Mit91* for SEMC ratio. M/B indicates mice heterozygous with both a MRL/MpJ and C57BL/6N allele. M/M indicates mice homozygous for the MRL/MpJ allele. Data represent mean ± SEM.

MRL/MpJ mice homozygous QTL on Chr 8 and heterozygous QTL on Chr 6 showed the highest values for OMC and SEMC densities, while QTL on Chr 6 had no effect on OMC number in mice with heterozygous QTL on Chr 8 (Fig. 5A, B). BMM mice carrying MRL/MpJ-type QTLs on Chrs 8 and 19 showed the highest values for SEMC density and SEMC ratio (Fig. 5C, D).

### Evaluation of SNPs and expression level of candidate genes

Genes on the *mcom1* locus associated with MC migration and chemotaxis were evaluated as candidates for the regulation of OMC density, because MCs were the only immune cell type present in the ovary of neonatal MRL/MpJ mice [21], [25]–[30]. SNPs of these genes were investigated in order to identify non-synonymous coding polymorphisms between MRL/MpJ and C57BL/6N mice using the SNP database available from the MPD. However, no non-synonymous coding polymorphisms were identified in MC migration- or chemotaxis-associated genes at *mcom1*, including Interleukin 15 (*Il15*), Matrix metallopeptidase 2 (*Mmp2*), Metallothionein 1–4 (*Mt1*–*Mt4*), Chemokine (C-C motif) ligand 17 (*Ccl17*), *Ccl22*, and Chemokine (C-X3-C motif) ligand 1 (*Cx3cl1*). In contrast, no genes associated with the MC migration or chemotaxis were reported at *mcom2.*

**Figure 6:**
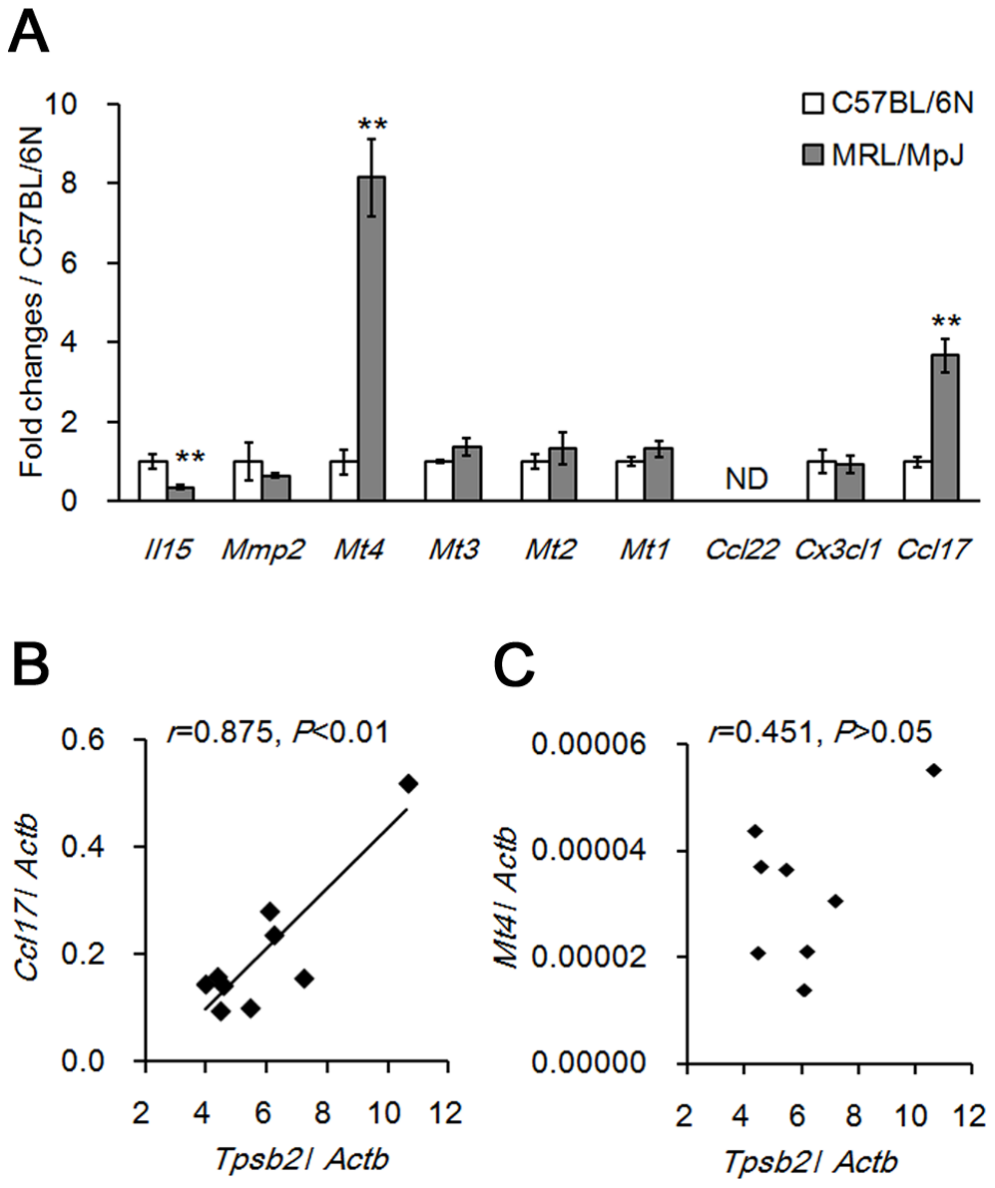
Candidate genes regulating the appearance of neonatal ovarian mast cells. (A) Quantitative real-time PCR analyses of candidate gene expression at postnatal day 0. Significant differences were analysed using the Mann-Whitney *U* test. **P < 0.01; ND, not detected. (B) Correlation between *Tpsb2* and *Ccl17* (Pearson’s correlation test, n = 9). (C Correlation between *Tpsb2* and *Mt4* (Pearson’s correlation test, n = 8).

Expression levels of these MC chemoattractant genes at P0 were compared between MRL/MpJ and C57BL/6N ovaries by qPCR. *Mt4* and *Ccl17* expression were significantly higher in MRL/MpJ than in C57BL/6N mice, while *Il15* level was significantly lower in MRL/MpJ (Fig. 6A). Expression levels of other candidate genes were comparable between the two strains (Fig. 6A). Expression of *Ccl17*, but not *Mt4*, was positively correlated with that of the OMC marker gene, tryptase beta 2 (*Tpsb2*) in P0 MRL/MpJ mouse ovary [21] (Fig. 6B, C).

## Discussion

### Influence of the maternal factors on OMC number

The appearance of OMCs varies according to mouse strain [21]. In neonatal MRL/MpJ mice, OMCs are more abundant than in other strains, including C57BL/6N, and they are distributed beneath the ovarian SE. In the present study, OMC density differed between MRLB6F1 and B6MRLF1, but was similar between F2 crosses, implying that maternally inherited mitochondrial haplotypes have no influence on this trait. These results led us to hypothesize that maternal factors during pregnancy could affect the number of OMCs in perinatal mice. In contrast, no difference was found in SEMC ratio between direct and reciprocal F1 hybrids. In addition, SEMC ratio was not affected by the progesterone-induced delayed parturition in both MRL/MpJ and C57BL/6N mice, indicating that MC distribution beneath the ovarian SE was not regulated by parental strains.

In MRL/MpJ fetuses with artificially delayed parturition, OMC density at E21.5 was significantly higher than in age-matched mice at P2. Fetuses were exposed to high doses of steroid hormones during late pregnancy, and both of estradiol and progesterone levels have been shown to affect MC migration [24]. Although plasma levels of these hormones were measured as putative maternal factors affecting OMC number, no differences were detected between MRL/MpJ and C57BL/6N mice at E18.5 of normal pregnancy. Taken together, these findings indicate that maternal factors during pregnancy influences OMC number by altering the fetal environment through factors other than plasma steroid hormone levels.

### Genetic regulation of OMC number

Because both the abundance of OMCs and their distribution beneath the ovarian SE were phenotypes unique to MRL/MpJ mice, three parameters were selected for QTL analysis: OMC and SEMC densities, as well as SEMC ratio. A high OMC density was correlated with the MRL/MpJ-type *D8Mit248*–*D8Mit312* on Chr 8 (*i.e.*, *mcom1* locus), whereas SEMC ratio was linked to a second locus, MRL/MpJ-type *D8Mit86*–*D8Mit89* on Chr 8 (*i.e.*, *mcom2*). These results clearly demonstrate that OMC abundance and distribution are regulated by independent genetic factors derived from *mcom1* and *mcom2*, respectively.

In addition, *D6Mit74* and *D19Mit91*–*D19Mit33* loci showed epistatic interactions with the *mcom1*, and *mcom2*, respectively. The genotypes on these chromosomes affected the appearance of MCs only in mice carrying MRL/MpJ homozygosity at *mcom1* and *mcom2*. These results indicate that these loci on Chrs 6 and 19 are positive modifiers of *mcom1* and *mcom2*. QTL analysis using F2 progeny would be useful for identifying relevant loci on other chromosomes, and evaluating interactions between *mcom1* and *mcom2*.

### Candidate genes affecting OMC number

The results of the QTL analysis suggested that genes at the *mcom1* locus could be major determinants of OMC number. Based on differences in gene expression between MRL/MpJ and C57BL/6N mice, two possible candidate genes were highlighted in *mcom1*: *Mt4* at 46.26 cM, and *Ccl17* at 46.85 cM. In contrast, no genes associated with the MC migration or chemotaxis were located at *mcom2*, suggesting that other molecular mechanisms, such as non-coding RNA, could regulate MC distribution.

*Ccl17* overexpression increases the number of MCs in chronic contact hypersensitivity, and *Ccl17*-deficient mice have reduced numbers of MCs in the skin [29], [31]. In the P0 ovary, *Ccl17* expression was upregulated in MRL/MpJ mice compared to C57BL/6N, and was positively correlated with the expression of the MC marker gene *Tpsb2*, suggesting *Ccl17* as a strong candidate gene at the *mcom1* locus.

Metallothioneins (MTs), comprising four subfamilies designated MT1 to MT4 in mammals, have ∼85% homology to CCL17 at the amino acid level, and immune cells migrate chemotactically toward MT1 and MT2 [30]. MT4 is expressed exclusively in stratified squamous epithelia such as oral epithelia, oesophagus, and skin, where MCs are abundant [32], [33]. Interestingly, the expression of *Mt1* through *Mt4* was detected in the P0 ovary, but only *Mt4* was upregulated in MRL/MpJ mice relative to C57BL/6N, although no correlation was observed between the expression of *Mt4* and *Tpsb2*. Therefore, the overexpression of *Mt4* could induce MC migration in an all-or-none rather than a concentration-dependent manner.

Gene expression profiles in oocytes change dramatically during early follicular development, including immune response genes such as interleukins and chemokines [34]. We previously reported a correlation between MC number and early follicular development [21]. Therefore, *mcom1* and *mcom2* would regulate the early follicular development of neonatal mice by altering the appearance of MC.

In summary, the appearance of OMCs was found to be closely associated with both environmental factors and filial genetic backgrounds. The environmental factors influenced the number but not the distribution of OMCs, but both were influenced by the filial genetic background and regulated by *mcom1* and *mcom2*, respectively. The identification of genes responsible for the appearance of OMCs helps to clarify the mechanisms underlying organ- and age-specific recruitment of MCs, and the role of these cells in early follicular development.

## Supporting Information

Table S1 (DOC)

Table S2 (DOC)
