# Supplementary material for: Genomic Analysis of the Appearance of Ovarian Mast Cells in Neonatal MRL/MpJ Mice
Source: PLoS One. 2014 Jun 23;9(6):e100617. doi: 10.1371/journal.pone.0100617 (PMC4067331; doi:10.1371/journal.pone.0100617)
Supplement: Table S1 — (DOC) [file pone.0100617.s001.doc]

Table S1. Microsatellite markers used for genotyping.

| Marker | cM | Marker | cM | Marker | cM | Marker | cM |
| --- | --- | --- | --- | --- | --- | --- | --- |
| *D1Mit123* | 17.67 | *D6Mit138* | 1.81 | *D9Mit90* | 17.80 | *D15Mit111* | 13.02 |
| *D1Mit181* | 38.54 | *D6Mit159* | 12.36 | *D9Mit302* | 36.36 | *D15Mit156* | 32.19 |
| *D1Mit191* | 52.66 | *D6Mit223* | 21.81 | *D9Mit76* | 50.18 | *D15Mit245* | 48.21 |
| *D1Mit107* | 70.19 | *D6Mit74* | 23.70 | *D9Mit18* | 71.49 |  |  |
| *D1Mit403* | 81.63 | *D6Mit316* | 27.41 |  |  | *D16Mit131* | 3.41 |
|  |  | *D6Mit188* | 32.53 | *D10Mit166* | 2.06 | *D16Mit59* | 26.86 |
| *D2Mit369* | 24.51 | *D6Mit10* | 52.75 | *D10Mit42* | 39.72 | *D16Mit140* | 40.30 |
| *D2Mit249* | 54.07 | *D6Mit194* | 62.90 | *D10Mit134* | 54.72 | *D16Mit106* | 57.68 |
| *D2Mit340* | 73.59 |  |  | *D10Mit271* | 72.31 |  |  |
| *D2Mit456* | 88.99 | *D7Mit178* | 2.02 |  |  | *D17Mit113* | 8.14 |
| *D2Mit148* | 100.49 | *D7Mit82* | 32.76 | *D11Mit62* | 5.78 | *D17Mit139* | 27.40 |
|  |  | *D7Mit321* | 53.57 | *D11Mit130* | 27.28 | *D17Mit119* | 38.15 |
| *D3Mit182* | 21.73 | *D7Mit105* | 70.29 | *D11Mit212* | 54.34 | *D17Mit221* | 59.77 |
| *D3Mit244* | 35.01 |  |  | *D11Mit48* | 82.96 |  |  |
| *D3Mit158* | 48.13 | *D8Mit4* | 18.89 |  |  | *D18Mit177* | 21.39 |
| *D3Mit350* | 63.88 | *D8Mit226* | 23.05 | *D12Mit136* | 13.00 | *D18Mit51* | 34.41 |
| *D3Mit129* | 80.49 | *D8Mit8* | 32.30 | *D12Mit158* | 38.14 | *D18Mit186* | 45.63 |
|  |  | *D8Mit343* | 39.33 | *D12Mit132* | 57.68 |  |  |
| *D4Mit235* | 3.57 | *D8Mit50* | 43.51 |  |  | *D19Mit80* | 18.24 |
| *D4Mit178* | 34.92 | *D8Mit248* | 44.99 | *D13Mit17* | 7.73 | *D19Mit19* | 34.08 |
| *D4Mit12* | 57.76 | *D8Mit312* | 47.12 | *D13Mit13* | 30.06 | *D19Mit91* | 40.53 |
| *D4Mit42* | 82.64 | *D8Mit242* | 50.07 | *D13Mit191* | 45.05 | *D19Mit33* | 51.76 |
|  |  | *D8Mit313* | 55.07 | *D13Mit260* | 63.73 |  |  |
| *D5Mit353* | 21.25 | *D8Mit86* | 56.18 |  |  | *DXMit166* | 28.26 |
| *D5Mit197* | 32.92 | *D8Mit200* | 61.37 | *D14Mit11* | 6.33 | *DXMit25* | 36.78 |
| *D5Mit240* | 53.24 | *D8Mit89* | 62.93 | *D14Mit133* | 16.80 | *DXMit130* | 55.45 |
| *D5Mit168* | 76.15 | *D8Mit42* | 74.46 | *D14Mit141* | 24.28 | *DXMit186* | 76.75 |
|  |  |  |  | *D14Mit37* | 33.21 |  |  |
|  |  |  |  | *D14Mit266* | 64.86 |  |  |
