# Supplementary material for: Genomic Analysis of the Appearance of Ovarian Mast Cells in Neonatal MRL/MpJ Mice
Source: PLoS One. 2014 Jun 23;9(6):e100617. doi: 10.1371/journal.pone.0100617 (PMC4067331; doi:10.1371/journal.pone.0100617)
Supplement: Table S2 — (DOC) [file pone.0100617.s002.doc]

Table S2. Gene-specific primer sequences and expected product sizes.

| Gene | Forward Primer (5’-3’) | Reverse Primer (5’-3’) | Product size | Accession # |
| --- | --- | --- | --- | --- |
| *Il15* | CATCCATCTCGTGCTACTTGTGTT | CATCTATCCAGTTGGCCTCTGTTT | 126 bp | NM_008357 |
| *Mmp2* | GGCTGGAACACTCTCAGGAC | CGATGCCATCAAAGACAATG | 213 bp | NM_008610 |
| *Mt4* | CTCAGCCTCCCTTTCTTAGC | ATCTCTCCCGCTGCCATAG | 253 bp | NM_008631 |
| *Mt3* | CCGGGAGGAACCAAGCTAC | GGGACACCCAGCACTATTTAC | 293 bp | NM_013603 |
| *Mt2* | TCTTCAAACCGATCTCTCGTC | GGCTAGGCTTCTACATGGTCT | 289 bp | NM_008630 |
| *Mt1* | GCTGTCCTCTAAGCGTCACC | GCTGGGTTGGTCCGATACT | 324 bp | NM_013602 |
| *Ccl22* | CCTGACGAGGACACATAACATC | TGGCAGAAGAATAGGGCTTG | 380 bp | NM_009137 |
| *Cx3cl1* | ATTGTCCTGGAGACGACACAG | TTGCCACCATTTTTAGTGAGG | 119 bp | NM_009142 |
| *Ccl17* | GAGTGCTGCCTGGATTACTTC | CGTCTCCAAATGCCTCAGC | 215 bp | NM_011332 |
| *Tpsb2* | CGACATTGATAATGACGAGCCTC | ACAGGCTGTTTTCCACAATGG | 80 bp | NM_010781 |
| *Actb* | TGTTACCAACTGGGACGACA | GGGGTGTTGAAGGTCTCAAA | 165 bp | NM_007393 |
